# Odontogenic Chronic Rhinosinusitis: Structured Histopathology Evidence in Different Patho-Physiological Mechanisms

**DOI:** 10.3390/biomedicines10112768

**Published:** 2022-10-31

**Authors:** Giuseppe Brescia, Lara Alessandrini, Christian Bacci, Guido Bissolotti, Marny Fedrigo, Giacomo Contro, Samuele Frasconi, Maria Grazia Boccuto, Arianna Calcavecchia, Anna Chiara Frigo, Umberto Barion, Stefano Fusetti, Annalisa Angelini, Gino Marioni

**Affiliations:** 1 Otolaryngology Section, Department of Neuroscience DNS, University of Padova, Via Giustiniani 2, 35128 Padova, Italy; 2 Surgical Pathology and Cytopathology Unit, Department of Medicine-DIMED, University of Padova, Via Giustiniani 2, 35128 Padova, Italy; 3 Clinical Dentistry, Department of Neuroscience DNS, University of Padova, Via Giustiniani 2, 35128 Padova, Italy; 4 Maxillo-Facial Surgery Unit, Department of Neuroscience DNS, University of Padova, Via Giustiniani 2, 35128 Padova, Italy; 5 Department of Cardiac-Thoracic-Vascular Sciences and Public Health, University of Padova, Via Giustiniani 2, 35128 Padova, Italy

**Keywords:** odontogenic chronic rhinosinusitis, structured histopathology, radicular cyst, dental implant, endoscopic sinus surgery

## Abstract

An increased odontogenic chronic rhinosinusitis (oCRS) occurrence rate has quite recently been reported, likely due to an intensification of conservative dental surgery and implantology. The main aim of the study was to report for the first time the structured histopathological characteristics of the surgical specimens of oCRS. Possible associations between histopathological features and oCRS patho-physiological mechanisms were also evaluated. Structured histopathology features were investigated in the sinonasal mucosa tissue of 42 consecutive oCRS patients. Mean tissue eosinophil counts were significantly different between oCRS with radicular cysts, dental implants, or other dental diseases (*p* = 0.0118): mean tissue eosinophil count was higher in oCRS with dental implants. Sub-epithelial edema score and squamous metaplasia presence were significantly different when comparing the above-mentioned sub-cohorts of oCRS (*p* = 0.0099 and *p* = 0.0258). In particular, squamous metaplasia was more present in oCRS cases with radicular cysts than in those with a dental implant (*p* = 0.0423). Fibrosis presence was significantly different comparing the three sub-cohorts of oCRS (*p* = 0.0408), too. This preliminary evidence supports the hypothesis that: (i) structural histopathology can become a useful tool for clinic-pathological practice in diagnostic, therapeutic, and prognostic terms in CRS; (ii) that oCRS, as CRS in general, is a histo-pathologically heterogeneous disease; (iii) oCRS resulting from dental implants disorders can frequently be characterized as a CRS with a rich tissue eosinophilic component.

## 1. Introduction

Chronic rhinosinusitis (CRS) is a multifactorial inflammatory disease of the nasal cavities and paranasal sinuses. Odontogenic CRS (oCRS) as a separate entity was first described in 1943 [1]. An increased oCRS occurrence rate has been reported quite recently, likely due to an increase in conservative dental surgery and implantology procedures [2,3,4]. An odontogenic process is detected in about 10–40% of cases of maxillary sinusitis and up to 75% of unilateral maxillary sinusitis [5], but despite this prevalence, odontogenic origins of sinusitis are still frequently misdiagnosed. From a patho-physiological viewpoint, oCRS develops from a dental infection spreading to the maxillary sinus through the mucoperiosteum (Schneiderian membrane) [6]. These infections can evolve into chronic exudative sinusitis or chronic polypoid sinusitis, which, in addition to the maxillary sinus, can involve other adjacent paranasal sinuses. Primary causes of oCRS include: i. dental caries leading to pulpitis and pulp necrosis, ii. dental abscesses, and iii. periodontal diseases that may result in a secondary infection of the dental pulp [6,7]. Peri-apical inflammation combined with the release of bacterial factors promotes tissue degradation and Schneiderian’s membrane perforation [7]. The pulp necrosis and loss of the biological barrier, which follows carious lesions or dental traumas, can lead to the formation of a granuloma and, subsequently, to an inflammatory radicular cyst [8]. Iatrogenic causes include: i. root canal therapy (migration into the maxillary sinus of endodontic cement or materials such as gutta-percha or broken instruments left in the root, ii. tooth extraction, iii. enucleation of cysts and granulomas, iv. maxillary osteotomies, v. dental implantation procedures [3,4,6,7,9], vi. bone infections due to the lifting of the maxillary sinus’ floor (in implant rehabilitations or, more rarely, during grafting procedures or periodontal debridement) [10,11,12], and vii. dental implants displacement in the paranasal sinuses [3,4].

Nowadays, in routine practice, a conventional histopathological approach on surgical samples offers limited information on the heterogeneous pathogenic mechanisms underlying CRS, but a potential role of structured histopathological profiling for CRS has begun to attract attention [13,14,15,16]. Structured histopathological examination of CRS could be a necessary step in efforts to establish CRS pathogenesis. The main aim of this study was to report in detail for the first time the structured histopathological characteristics of surgical specimens of oCRS patients who underwent sinus surgery. Possible associations between histopathological features and oCRS patho-physiological mechanisms were also evaluated.

## 2. Materials and Methods

### 2.1. Patients

The present study is a retrospective clinical investigation. No experimental diagnostic or therapeutic procedures have been applied; the procedures carried out are standardized clinical procedures in our daily practice. The study was conducted in accordance with the principles of the Helsinki Declaration. All patients signed a detailed informed consent form regarding the processing and publication of their data. They consented to “the use of their clinical data for scientific research purposes in the medical, biomedical and epidemiological fields, also in order to be recalled in the future for follow-up needs”. Data were examined in agreement with the Italian privacy and sensitive data laws and the internal regulations of the University Hospital of Padova.

The study retrospectively assessed 42 consecutive adult patients suffering from oCRS and treated from 2014 to 2020: 24 patients (57.1%) were male. The mean age at surgery was 54.0 ± 12.1 years (median 55 years). The teeth involved in sinusitis were the first molar (ten cases), the second premolar (six cases), but also the canine, the first premolar, the second molar (four cases each), and the third molar (two cases). Data were missing for 12 patients. The mean duration of oCRS symptoms was 17.6 ± 19.0 months (median 12 months). Two of the patients had a diagnosis of asthma, two of allergies, and three had both diagnoses. Table 1 reports the cohort’s main demographic and clinical features.

All patients underwent rigid nasal endoscopy (4 mm, 0°, and 30° telescopes) under local anesthesia. Furthermore, a radiological evaluation (both orthopantomography and paranasal sinuses computerized tomography [CT] scan) was performed to evaluate the presence of sino-nasal inflammation or anatomical alterations, such as nasal septum deviation or osteomeatal complex (OMC) alterations. The CT scan was also relevant to show the concomitant presence of odontogenic cysts (or other odontogenic disorders) and subclinical bone fistulas.

Our patients were classified into three groups based on etiology: (i) radicular cysts, (ii) dental implants, (iii) other dental diseases such as caries and periodontitis leading to secondary pulpitis, dental abscesses, and iatrogenic causes (previous root canal therapies or tooth extractions).

Table 2 summarizes the phenotype (polypoid/non-polypoid), lateralization, and CT score [17] of the considered oCRS series.

### 2.2. Treatment

Based on the clinical and radiological findings, each case was discussed in a multidisciplinary setting to decide the appropriate surgical approach to treat the oCRS. Sixteen patients underwent surgery through a transoral approach, five patients underwent a transnasal endoscopic approach, and 21 underwent a combined transoral/transnasal approach.

#### 2.2.1. Transoral Approach

Conscious sedation was induced with oral chlormethyldiazepam 30–60 min prior to the scheduled treatment and then intraoperatively with intra-venous diazepam or midazolam [17]. Local anesthesia of the affected maxilla was performed with a nerve block of the maxillary nerve along the greater palatine canal and vestibular anesthesia of the middle and/or posterior superior alveolar nerve. A full-thickness muco-periosteal vestibular flap was then prepared, with releasing incisions medial and distal to the oro-antral communication (already present or induced by a dental extraction, implant, or a residual root inside the antrum) and bone defect. Skeletonization, ostectomy, and osteoplasty were then performed. A toilette of the maxillary sinus was then performed by negative suction and direct vision of the sinus itself. The oro-antral communication was closed using a buccal fat pad flap, which was secured with an absorbable.

#### 2.2.2. Transnasal Endoscopic Approach

It is common knowledge that most oCRS cases can be treated by a trans-nasal endoscopic approach, especially with the introduction of modern techniques [18,19,20]. In our series, the endoscopic procedure was performed under general anesthesia using a 4-mm rigid endoscope (0° or 45°). A sinus surgery was performed to remove nasal polyps or clean sinuses and/or correct anatomical alterations such as nasal septal deviation (9 patients) or the presence of a concha bullosa (7 cases). When the OMC was clearly accessible, an uncinectomy and middle antrostomy were performed. With the aid of an angled endoscope, the maxillary cavity was then cleaned through the middle meatus and any foreign bodies were removed [3,4]. After surgery, the nasal cavity was packed with an 8 × 1 cm non-inflatable, gel-coated intranasal splint (Rapid Rhino^TM^, Smith & Nephew Inc., Austin, TX, USA) to control bleeding. The nasal pack was removed on the first or second postoperative day.

In cases where a single approach was not sufficient to ensure adequate removal of the inflammation, it was decided to treat sinonasal disease and alveolar bone involvement at the same time with a combined oronasal approach.

The tissue sample collected from the maxillary sinus during surgery was sent for histopathological examination.

### 2.3. Histopathological Investigations

A dedicated head and neck pathologist (L.A.) and a general pathologist (M.F.) blindly analyzed all hematoxylin and eosin (H&E)-stained slides available from each surgical specimen under a light microscope to assess and score thirteen histopathological variables according to the method applied previously by our group [15] and by others [14].

Slides were examined at low-power magnification (×40) to identify the most representative fields for each histological feature considered. Selected areas were then examined at high-power magnification (100× or 400×) and scored. In case of disagreement on the diagnosis, the slides were reviewed with a multi-head microscope by the two pathologists until a consensus was met.

### 2.4. Statistical Analysis

The statistical analyses were performed with SAS 9.4 (SAS Institute Inc., Cary, NC, USA) for Windows.

Categorical variables were summarized with the number and percentage of cases in each category, quantitative ones with mean and standard deviation (SD), median, and range. Comparison of histopathological features across the sub-cohorts of oCRS patients was performed with Fisher’s exact test in the case of categorical variables, with the Kruskal–Wallis test for those quantitative. The between-group differences were estimated with a 95% confidence interval (CI) calculated with the asymptotic Hodges–Lehmann estimation for quantitative variables, with the exact binomial Clopper–Pearson method for binomial variables. The statistical significance was stated when *p* < 0.05.

## 3. Results

Structured histopathology was evaluated in 42 cases of oCRS. Table 3 (left column) summarizes the considered histopathological features. The mean eosinophil count in oCRS tissue was 7.2 ± 13.3 cells/5HPF (median 2.0; range 0.0–75.0).

### Structured Histopathology and oCRS Sub-Cohorts Stratified on Etiological Basis

**Figure 1:**
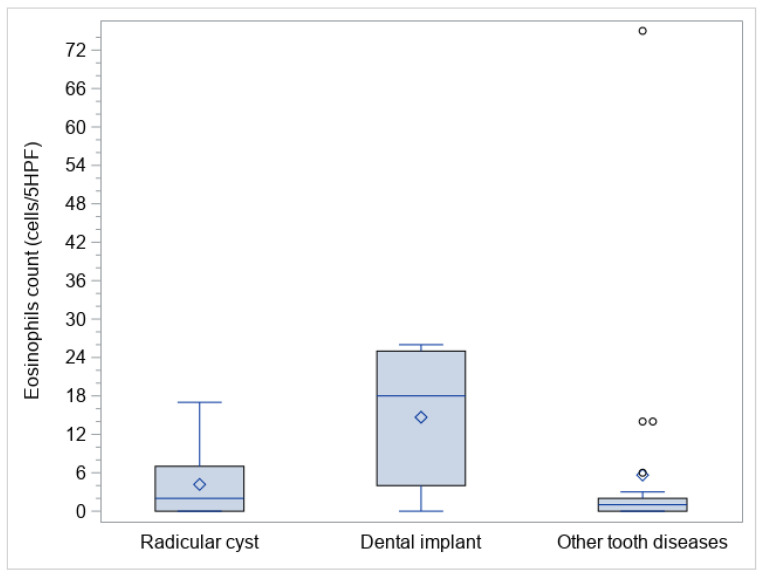
Box plots of eosinophil polyps tissue count in the evaluated sub-cohorts.

**Figure 2:**
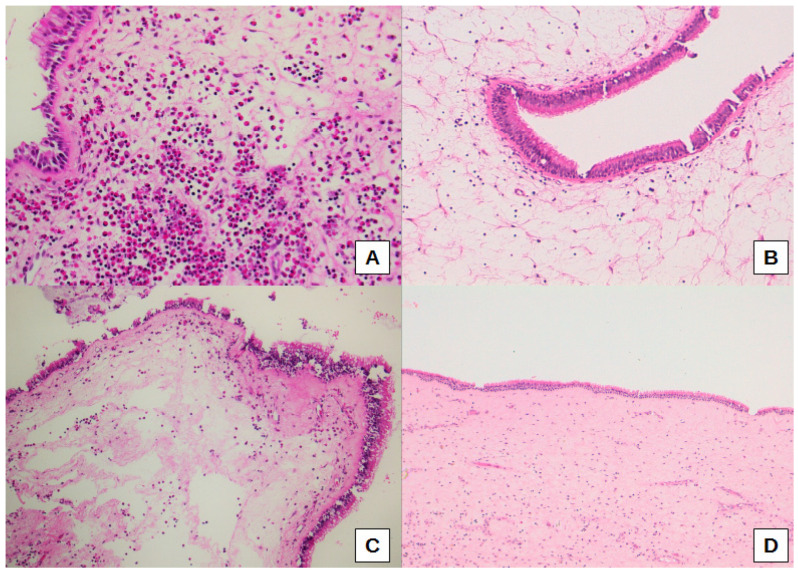
Representative histological images of a high eosinophil count with eosinophil aggregates in sub-epithelial connective tissue in a patient with a dental implant (**A**), a marked (**B**), and moderate (**C**) sub-epithelial edema, the latter associated with a high eosinophil count, in a case with radicular cyst. Normal mucosa for comparison (**D**). Original magnification: 200× (**A**), 100× (**B**,**C**), 50× (**D**).

Three sub-cohorts of oCRS were identified on an etiological basis: eleven patients (26.2%) had radicular cysts, nine patients (21.4%) had dental implants, and twenty-two (52.4%) other dental diseases. Table 3 reports the structured histopathological features stratified according to the above-mentioned oCRS sub-cohorts. Median tissue eosinophil counts were significantly different between the three sub-cohorts (*p* = 0.0118) (Figure 1). In particular, median tissue eosinophil count was higher in oCRS with dental implants than in those with other tooth diseases (difference of medians 11, 95% CI: 2; 22) (Table 3; Figure 2A).

**Figure 3:**
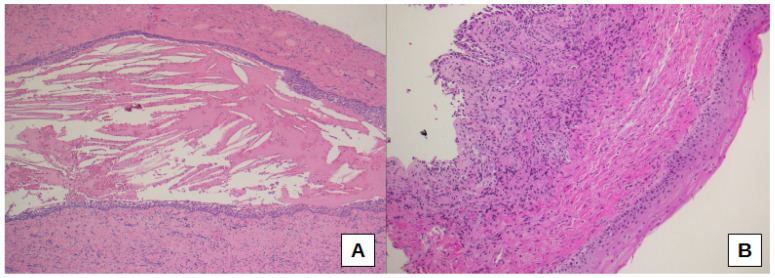
Squamous metaplasia is evident in a case of oCRS with radicular cyst (**A**,**B**); at the top left, normal epithelium could be seen (**B**). Original magnification: 50× (**A**), 100× (**B**).

Thus, sub-epithelial edema was significantly different when comparing the three sub-cohorts of oCRS (*p* = 0.0099). Sub-epithelial edema was lower in oCRS cases with other tooth diseases than in cases with radicular cyst (difference of proportions −27.3%, 95% CI: −61.0%; −4.1%) or dental implant (difference of proportion −33.3%, 95% CI −70.1%; −6.5%) (Figure 2B,C). Figure 2D shows normal mucosa for comparison. Moreover, squamous metaplasia presence was significantly different between the three sub-cohorts (*p* = 0.0258). In particular, squamous metaplasia was more present in oCRS cases with radicular cysts than in those with a dental implant (difference of proportion 54.5%, 95% CI 14.9%; 83.3%) (Figure 3A,B).

**Figure 4:**
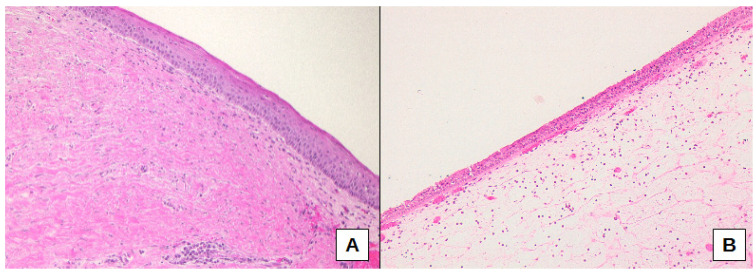
Connective tissue fibrosis, as well as epithelial squamous metaplasia, were more frequently present in cases with radicular cysts (**A**), whereas cases associated with dental implants showed sub-epithelial edema and normal pseudo-stratified columnar ciliated epithelium (**B**). Original magnification: 100× (**A**), 100× (**B**).

Furthermore, fibrosis presence was significantly different comparing the three sub-cohorts of oCRS (*p* = 0.0408). Fibrosis was less present in oCRS cases with dental implants than in cases with radicular cysts (difference of proportion −52.5%, 95% CI −82.7%; −8.1%) (Figure 4A,B).

**Figure 5:**
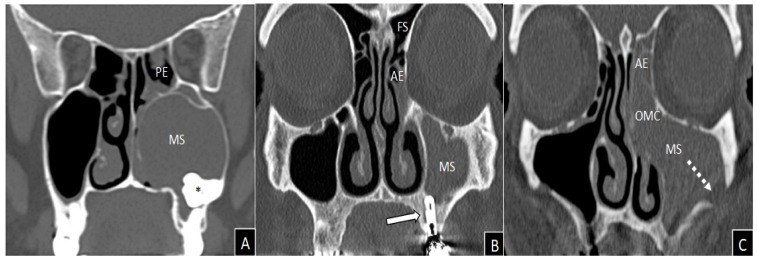
Coronal views of computed tomography imaging. Massive and homogeneous left maxillary sinus opacification in a non-eosinophilic polypoid oCRS (tissue eosinophils count 0/5HPF) caused by an included tooth (**asterisk**), which deforms the medial wall of the maxillary sinus causing obliteration of the ostiomeatal complex (**A**). Left anterior ethmoidal and maxillary sinuses inflammatory involvement in a patient with non-eosinophilic oCRS without polyps (tissue eosinophils count 10/5HPF) caused by dental implantation of element 2.6 (**white arrow**) (**B**). Massive inflammation of left maxillary and anterior ethmoid with obliteration of the ostiomeatal complex in a patient with oro-antral fistula (**white dashed arrow**) and non-polypoid eosinophilic oCRS (tissue eosinophils count 22/5HPF) caused by zygomatic implant (**C**). AE = anterior ethmoid; MS = maxillary sinus; OMC = ostiomeatal complex; PE = posterior ethmoid; FS = frontal sinus.

Figure 5 shows some CT pictures of oCRS characterized by different etiopathogenesis and histopathological morphology, in particular in terms of representation of the eosinophilic cytological component.

## 4. Discussion

The sinus epithelium is the primary barrier for physical, chemical, and immunologic stimuli; damaged epithelium plays a key role in driving tissue remodeling. Tissue remodeling in CRS is a process involving temporary or permanent changes [21,22]. To the best of our knowledge, there is little data regarding detailed histopathological features in the sinonasal mucosa of patients with oCRS [12,23]. The present study investigated sinonasal structured histopathology in terms of thirteen histopathological variables in sinonasal mucosa tissue of oCRS patients (see Table 3, column 1). Associations between these histopathological features and oCRS patho-physiological mechanisms were also analyzed.

Structured histopathology can provide relevant information for understanding oCRS because it considers not only the type of inflammatory cells but also their tendency to form aggregates and distribution in the stroma. This investigation’s main strength lies in the multidisciplinary setting in which all patients were diagnosed and treated, including the surgical procedures and the histopathological analysis evaluated by the same pathologists. Moreover, these analyses were not conducted on small biopsies but only on larger surgical specimens, allowing an accurate analysis of the different cells’ infiltration and distribution in the tissues, as previously reported in other inflammatory sinonasal disorders [24]. Despite initially being time-consuming, once a pathologist had completed the training practice, the use of structured histopathology did not increase the usual time to complete a routine pathology report. It could also be considered a cost-effective additional source of clinical information, as it does not require further laboratory techniques that could cause a diagnostic delay [15]. On the other hand, the study’s weaknesses are the retrospective setting and the limited number of patients involved.

Structured histopathological analysis of oCRS highlighted the presence of a high degree of inflammation (76.2%), mainly composed of plasma cells and lymphocytes, with a slight predominance of the former. Fibrosis was detected in nearly half of the cases, whereas basal membrane thickness, sub-epithelial edema, hyperplastic/papillary changes, mucosal ulceration, squamous metaplasia, and goblet cell hyperplasia were mostly absent. Fungal hyphae or spores were found only rarely.

When stratifying our oCRS cohort into three groups on an etiological basis (radicular cysts, dental implants, or other dental diseases), mean tissue eosinophil counts were significantly different. Interestingly, mean tissue eosinophil count was higher in oCRS with dental implants than in oCRS with radicular cysts or other tooth diseases. Although eosinophils have not been traditionally associated with oCRS, a similar finding was reported by Raman et al. [7], who identified an increased tissue eosinophilia in approximately 40% of oCRS specimens. Increased eosinophilia may contribute to the predisposition of a subset of patients with odontogenic lesions to develop oCRS. Eosinophils play a crucial role in immune homeostasis, both as effector immune cells engaged in host defense and as modulators of innate and adaptive immune responses [25]. An intricate eosinophil-centered signaling network that includes Th2 lymphocytes, B cells, and mast cells, as well as platelets and circulating cells residing at sites of inflammation, is activated under different inflammatory stimuli to ensure host protection from parasitic, fungal, bacterial, and viral infections. However, the same mechanism explains the development of tissue damage during infections, eosinophils diseases and/or cell subgroups related to eosinophils, as well as in hypersensitivity reactions and autoimmune diseases [26]. Tissue eosinophil aggregates are a sign of eosinophilic activation and may point to a more severe disease because eosinophils are the major effectors of host tissue damage because of their propensity to release highly charged basic proteins, which have multiple cytotoxic effects [27]. Only a few studies analyzed the histopathology of peri-implant mucositis. The inflammatory infiltrates occupied a large surface area with a high number of leukocytes and microvessel density [28]. Zitzmann et al. [29] experimentally induced peri-implant mucositis and indicated a significant increase in T-cell density in peri-implant tissues. Two studies reported the predominant existence of T-lymphocytes in the infiltrated connective tissue of peri-implant mucositis lesions [30,31]. Obădan et al. [32] reported a high number of B-lymphocytes in peri-implant mucositis, which was predominant in some areas of the *lamina propria*.

In the sub-cohort of oCRS patients with dental implants, in addition to a higher eosinophil count, the presence of edema of the mucosa was significantly higher compared to the cases with a radicular cyst or other dental pathologies. Eosinophilic CRS has been reported to be characterized more by edema and less by fibrosis in comparison with non-eosinophilic CRS. Activated eosinophils at sites of inflammation may contribute to increased vascular permeability and subsequent tissue edema in the sub-cohort with dental implants by releasing Vascular Endothelial Growth Factor, a vascular-endothelial-cell specific cytokine that mediates angiogenesis and vascular permeability [22,25]. Considering oCRS cases with dental implants, fibrosis was lower than in cases with radicular cysts; comprehensively, fibrosis presence was significantly different comparing the three sub-cohorts of oCRS, as it could be considered a long-term connective tissue response to damage. Although the onset of an implant-related fibrosis shares several features with normal wound healing, as in radicular cyst-related fibrosis, the nature of the implanted material has a profound impact on the progression of acute immune and repair reactions into chronic conditions [33]. This could partly explain the differences in fibrosis presence among oCRS groups [15,16]. Squamous metaplasia was more present in oCRS cases with radicular cysts than in those with a dental implant. In a clinic-pathological study of squamous metaplasia in CRS, Myniatt et al. [34] found that metaplasia was present in approximately 18% of routine CRS samples. Squamous metaplasia had a positive association with the severity of histologically observed inflammation but was not clinically related to the severity or chronicity of the disease.

## 5. Conclusions

This preliminary evidence, although still to be confirmed, supports the hypothesis that: (i) structural histopathology can become a useful tool for clinic-pathological practice in diagnostic, therapeutic, and prognostic terms in chronic rhinosinusitis; (ii) oCRS, as chronic rhinosinusitis in general, is an histo-pathologically heterogeneous disease; (iii) oCRS resulting from dental implants disorders can frequently be characterized as chronic rhinosinusitis with a rich tissue eosinophilic component.

## Figures and Tables

**Table 1:** oCRS series (42 cases): Main demographic and clinical features.

Main Features	No. of Cases (%)
**Sex**	
Male	24 (57.1)
Female	18 (42.9)
**Allergy/Asthma**	
None	35 (83.3)
Allergy	2 (4.8)
Asthma	2 (4.8)
Allergy and asthma	3 (7.1)
**Sinonasal polyps’ phenotype**	
No	21 (50.0)
Yes	21 (50.0)

**Table 2:** oCRS; phenotype (polypoid/non-polypoid), lateralization, CT score, and the statistical analysis evaluating the association with pathogenesis.

	Total (42 Cases)	Radicular Cyst (11 Cases)	Dental Implants (9 Cases)	Other Tooth Diseases (22 Cases)	*p*-Value
oCRS with nasal polyps	21 (50.0)	4 (36.4)	6 (66.7)	11 (50.0)	0.4375
oCRS without nasal polyps	21 (50.0)	7 (63.6)	3 (33.3)	11 (50.0)	
Unilateral polyposis	19 (90.5)	4 (100.0)	5 (83.3)	10 (90.9)	
Bilateral polyposis	2 (9.5)	0 (0.0)	1 (16.7)	1 (9.1)	
CT score					
Mean (SD)	4.7 (3.4)	4.5 (2.6)	5.8 (1.9)	4.1 (4.2)	0.3423
Median (Range)	4.0 (1.0–12.0)	5.0 (1.0–7.0)	5.5 (4.0–9.0)	2.0 (1.0–12.0)	

**Table 3:** Structured histopathological features stratified according to the etiologically-based oCRS sub-cohorts.

	No. of Cases (%)	Radicular Cyst (1) No. of Cases = 11	Dental Implant (2) No. of Cases = 9	Other Tooth Diseases (3) No. of Cases = 22	Overall *p*-Value	Difference (95% CI) 2 vs. 1	Difference (95% CI) 3 vs. 1	Difference (95% CI) 2 vs. 3
**Degree of inflammation**								
0 or 1	10 (23.8)	3 (27.3)	1 (11.1)	6 (27.3)	0.7106			
2 or 3	32 (76.2)	8 (72.7)	8 (88.9)	16 (72.7)		16.2 (−25.1; 52.3)	0.0 (−30.7; 36.3)	16.2 (−22.8; 43. 2)
**Eosinophils count (cells/5HPF)**								
Mean (SD)	7.2 (13.3)	4.2 (5.3)	14.7 (10.7)	5.6 (16.0)	0.0118			
Median (Range)	2.0 (0.0–75.0)	2.0 (0.0–17.0)	18.0 (0.0–26.0)	1.0 (0.0–75.0)		10 (0; 22)	0 (−4; 1)	11 (2; 22)
**Eosinophil aggregates**								
No	41 (97.6)	11 (100.0)	9 (100.0)	21 (95.5)	1.0000			
Yes	1 (2.4)	0 (0.0)	0 (0.0)	1 (4.5)		-	4.5 (−24.5; 23.3)	−4.5 (−23.2; 30.0)
**Neutrophil infiltrate**								
No	16 (38.1)	5 (45.5)	2 (22.2)	9 (40.9)	0.5873			
Yes	26 (61.9)	6 (54.5)	7 (77.8)	13 (59.1)		23.3 (−21.3; 62.6)	4.6 (−31.1; 40.6)	18.7 (−21.5; 49.1)
**Basal membrane thickening**								
0 or 1	37 (88.1)	8 (72.7)	9 (100.0)	20 (90.9)	0.1909			
2 or 3	5 (11.9)	3 (27.3)	0 (0.0)	2 (9.1)		−27.3 (−61.0; 9.9)	−18.2 (−51.8; 9.9)	−9.1 (−30.0; 25.5)
**Subepithelial edema**								
0 or 1	36 (85.7)	8 (72.7)	6 (66.7)	22 (100.0)	0.0099			
2 or 3	6 (14.3)	3 (27.3)	3 (33.3)	0 (0.0)		6.1 (−32.2; 48.3)	−27.3 (−61.0; −4.1)	33.3 (6.5; 70.1)
**Hyperplastic—papillary changes**								
No	33 (78.6)	9 (81.8)	6 (66.7)	18 (81.8)	0.6151			
Yes	9 (21.4)	2 (18.2)	3 (33.3)	4 (18.2)		15.1 (−25.7; 54.8)	0.0 (−33.7; 27.3)	15.1 (−17.6; 52.0)
**Mucosal ulceration**								
No	32 (76.2)	6 (54.5)	7 (77.8)	19 (86.4)	0.1327			
Yes	10 (23.8)	5 (45.5)	2 (22.2)	3 (13.6)		−23.2 (−62.6; 21.3)	−31.8 (−64.5; 1.8)	8.6 (−20.2; 47.4)
**Squamous metaplasia**								
No	30 (71.4)	5 (45.5)	9 (100.0)	16 (72.7)	0.0258			
Yes	12 (28.6)	6 (54.5)	0 (0.0)	6 (27.3)		−54.5 (−83.3; −14.9)	−27.3 (−61.0; 11.1)	−27.3 (−50.5; 9.3)
**Fibrosis**								
No	22 (52.4)	4 (36.4)	8 (88.9)	10 (45.5)	0.0408			
Yes	20 (47.6)	7 (63.6)	1 (11.1)	12 (54.5)		−52.5 (−82.7; −8.1)	−9.1 (−42.6; 28.7)	−43.4 (−70.1; 2.1)
**Fungal elements**								
No	39 (92.9)	10 (90.9)	9 (100.0)	20 (90.9)	1.0000			
Yes	3 (7.1)	1 (9.1)	0 (0.0)	2 (9.1)		−9.1 (−41.3; 25.2)	0.0 (−32.9; 22.9)	−9.1 (−30.0; 25.5)
**Charcot–Leyden crystals**								
No	42 (100.0)	11 (100.0)	9 (100.0)	22 (100.0)	-			
Yes	0 (0.0)	0 (0.0)	0 (0.0)	0 (0.0)				
**Globet cells hyperplasia**								
0 or 1	30 (71.4)	7 (63.6)	5 (55.6)	18 (81.8)	0.2875			
2 or 3	12 (28.6)	4 (36.4)	4 (44.4)	4 (18.2)		8.1 (−38.0; 50.2)	−18.2 (−53.1; 13.6)	26.2 (−9.6; 61.9)

## Data Availability

The datasets generated and analyzed during the current study are available on reasonable request.
